# Can Dynamic Whole-Body FDG PET Imaging Differentiate between Malignant and Inflammatory Lesions?

**DOI:** 10.3390/life12091350

**Published:** 2022-08-30

**Authors:** Stephan Skawran, Michael Messerli, Fotis Kotasidis, Josephine Trinckauf, Corina Weyermann, Ken Kudura, Daniela A. Ferraro, Janique Pitteloud, Valerie Treyer, Alexander Maurer, Martin W. Huellner, Irene A. Burger

**Affiliations:** 1 Department of Nuclear Medicine, University Hospital Zurich, 8091 Zurich, Switzerland; 2 Faculty of Medicine, University of Zurich, 8006 Zurich, Switzerland; 3 GE Healthcare, Waukesha, WI 53188, USA; 4 Claraspital, 4058 Basel, Switzerland; 5 Department of Nuclear Medicine, Kantonsspital Baden, 5404 Baden, Switzerland

**Keywords:** dynamic whole-body positron emission tomography, infection, fluorodeoxyglucose, Patlak, oncologic imaging, molecular imaging, FDG PET/CT

## Abstract

*Background:* Investigation of the clinical feasibility of dynamic whole-body (WB) [^18^F]FDG PET, including standardized uptake value (SUV), rate of irreversible uptake (Ki), and apparent distribution volume (Vd) in physiologic tissues, and comparison between inflammatory/infectious and cancer lesions. *Methods:* Twenty-four patients were prospectively included to undergo dynamic WB [^18^F]FDG PET/CT for clinically indicated re-/staging of oncological diseases. Parametric maps of Ki and Vd were generated using Patlak analysis alongside SUV images. Maximum parameter values (SUV_max_, Ki_max_, and Vd_max_) were measured in liver parenchyma and in malignant or inflammatory/infectious lesions. Lesion-to-background ratios (LBRs) were calculated by dividing the measurements by their respective mean in the liver tissue. *Results:* Seventy-seven clinical target lesions were identified, 60 malignant and 17 inflammatory/infectious. Ki_max_ was significantly higher in cancer than in inflammatory/infections lesions (3.0 vs. 2.0, *p* = 0.002) while LBRs of SUV_max_, Ki_max_, and Vd_max_ did not differ significantly between the etiologies: LBR (SUV_max_) 3.3 vs. 2.9, *p* = 0.06; LBR (Ki_max_) 5.0 vs. 4.4, *p* = 0.05, LBR (Vd_max_) 1.1 vs. 1.0, *p* = 0.18). LBR of inflammatory/infectious and cancer lesions was higher in Ki_max_ than in SUV_max_ (4.5 vs. 3.2, *p* < 0.001). LBRs of Ki_max_ and SUV_max_ showed a strong correlation (Spearman’s rho = 0.83, *p* < 0.001). *Conclusions:* Dynamic WB [^18^F]FDG PET/CT is feasible in a clinical setting. LBRs of Ki_max_ were higher than SUV_max_. Ki_max_ was higher in malignant than in inflammatory/infectious lesions but demonstrated a large overlap between the etiologies.

## 1. Introduction

Positron emission tomography (PET) is a powerful imaging modality for the non-invasive assessment of different physiological and pathological processes at a molecular level [1,2]. With 2-[^18^F]fluoro-2-deoxy-D-glucose ([^18^F]FDG), a glucose analog, PET can be used to image glucose metabolism, and thus metabolically active processes, such as malignant or inflammatory diseases [3].

Current [^18^F]FDG PET diagnostics in clinical routine use static imaging after a certain uptake period. The qualitative visual assessment of the images is often aided by the use of semi-quantitative indices (i.e., measurement of standardized uptake value—SUV [4]). However, such analyses suffer from inherent variability due to a multitude of acquisition-related and physiological issues, limiting the full diagnostic potential of PET [5,6]. Indeed, the specificity of SUV measurements may be impaired by the fact that non-specific uptake (e.g., unmetabolized tissue [^18^F]FDG, and fractional blood volume) is not negligible towards earlier scan times or in tissues with high blood fraction components [7]. Furthermore, the area under the curve of the plasma tracer activity concentration is very often not proportional to the injected dose due to extravasation, injection residual, weight inaccuracy, or plasma clearance changes from excretion [8]. More sophisticated indexes than SUVs, which try to overcome some of these drawbacks have been proposed. An SUV ratio normalized to the blood pool could provide a more reliable surrogate in that respect. However, certain parameter assumptions might not always be fulfilled across the scanned population and might be tracer-specific [9,10,11,12,13].

During the last few years, several studies have highlighted the diagnostic role of kinetic modeling following the dynamic acquisition of PET data, which can provide additional surrogate parameters more closely related to tumor characteristics, including less inherent assumptions [14]. At the same time, the kinetic analysis may assist in the therapy response assessment by reflecting more accurately changes after therapy [15,16,17,18]. While routine static [^18^F]FDG PET/CT imaging produces a single SUV image, dynamic [^18^F]FDG PET/CT acquisition, followed by appropriate kinetic analysis, produces additional parametric images, each of which conveys a different physiological meaning. While SUV images sum up the entire [^18^F]FDG signal, parametric images could allow for differentiation between free [^18^F]FDG and [^18^F]FDG retained in glucose-consuming tissue.

Extending dynamic imaging from single bed position to whole-body dynamic (WBD) acquisition poses some specific requirements on data acquisition (repeated table passes over the same axial location) and dedicated acquisition protocols coupled with kinetic models [19]. Patlak analysis allows the estimation of macro-parameter images derived from the combination of individual microparameters, namely the irreversible uptake rate (Ki) representing the estimated [^18^F]FDG influx into the tissue, and the apparent distribution volume (Vd) representing the volume of free [^18^F]FDG in the reversible compartments and blood volume [20].

The use of dedicated WBD acquisition protocols for Patlak analysis allows not only to extend the effective dynamic field of view (FOV) but also enables the non-invasive estimation of an image-derived input function (IDIF). Instead of individual estimation of IDIF, population approaches have also been used extensively in dynamic [^18^F]FDG studies successfully. However, particularly in post-therapeutic settings, these might struggle to accurately represent the patient-specific input function, due to potential changes in plasma clearance, varying injection protocols, or due to IDIF scaling sensitivity at early and late times post-injection [21,22,23].

With growing computational power, increasing the sensitivity of scanners, and improved image reconstruction algorithms, WBD PET has become feasible [19,24,25,26]. Despite promising results proposing a role for Ki [27,28,29], WBD PET is still lacking widespread clinical adoption.

Accordingly, the aims of our study were to demonstrate the clinical feasibility of WBD acquisition and analysis. The study additionally aimed to quantify WBD PET parameters in normal tissue and compare the parameters derived from benign infectious and inflammatory lesions to cancer lesions in a cohort of oncologic patients.

## 2. Materials and Methods

### 2.1. Study Design and Population

Patients included in this study were examined between September 2018 and September 2019, and are part of an ongoing prospective single-center trial on WBD PET. Patients with a variety of clinical indications were included if they were scheduled for the first [^18^F]FDG PET/CT in the morning and agreed to spend the uptake time on the scanner. The local ethics committee approved the study (Cantonal Ethics Committee Zurich, Switzerland, BASEC-N^0^ 2018-01012, approved on 17 August 2018). All patients gave written informed consent for WBD imaging in addition to a clinically indicated PET/CT in the same session. The study was conducted in compliance with ICH-GCP rules and the Declaration of Helsinki.

### 2.2. WBD PET Acquisition and Image Reconstruction

Examinations were performed on a silicon photomultiplier (SiPM)-based 25 cm axial FOV PET/CT scanner (GE Discovery MI, GE Healthcare, Waukesha, WI, USA). A standardized clinical protocol with a body mass index (BMI) adapted [^18^F]FDG dosage protocol was used as previously described [30]: 1.5 MBq/kg body weight [^18^F]FDG was injected for patients with a BMI of < 20 kg/m^2^; 2 MBq/kg body weight for patients with a BMI of 20–24.5 kg/m^2^; and 3.1 MBq/kg body weight for patients with a BMI > 24.5 kg/m^2^, never exceeding a maximum injected activity of 320 MBq [^18^F]FDG. Injections were performed using an automated integrated dispensing/infusion system (MEDRAD^®^ Intego). Participants fasted for at least 4 h before [^18^F]FDG injection. Data acquisitions were split into 2 parts with the WBD acquisition taking place during the first 60 min post-injection, followed by the clinical routine acquisition at 60 + 10 min post-injection (with a break in between if the patient needed to void).

A CT scan obtained from the vertex of the skull to the mid-thighs or feet (e.g., in the case of lower extremity melanoma) was used for anatomical localization as well as attenuation correction. The CT scan was acquired using automated tube dose modulation (range 15–100 mA) with 120 kV.

WBD acquisitions included an initial single bed continuous dynamic scan over the heart for 10 ± 4 min [12 × 5 s, 4 × 10 s, 8 × 25 s, (5 ± 4) × 60 s] followed by consecutive temporally non-continuous head to thighs (5 ± 1 bed positions) unidirectional acquisitions over the same effective axial FOV for 50 ± 10 min up to a total scan duration of 60 ± 10 min post-injection (11 ± 3 whole body passes, 35 s/bed).

Acquisitions across the patients’ cohort were performed in both directions but always kept the same across whole body passes within each patient (unidirectional), to maintain evenly spaced temporal gaps between successive dynamic frames. 

After the WBD acquisition, the standard clinical acquisition was carried out, which was then used in the clinical routine.

Dynamic PET data from the blood pool and WBD acquisitions as well as the clinical static images were reconstructed using penalized likelihood reconstruction (Q.Clear, GE Healthcare, Waukesha, WI, USA) with a β-value of 450 on a 256 × 256 image matrix with a 2.79 mm slice thickness.

### 2.3. Generation of Patlak Parametric Images

**Figure 1:**
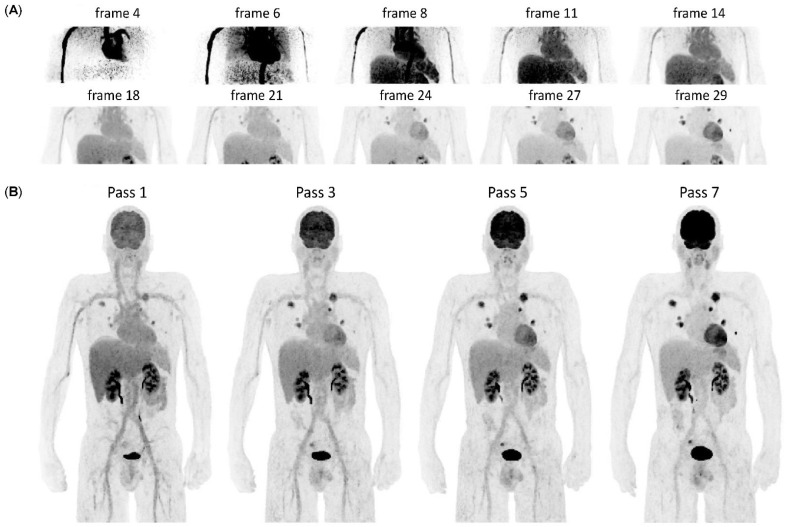
Examples of blood pool single bed dynamic (**A**) and head-to-thighs dynamic (**B**) reconstructed images (injected activity: 2 MBq/kg body weight—156 MBq in total) from representative dynamic frames, illustrating the original imaging data used for generating parametric maps.

Parametric images of Ki and Vd were generated following Patlak graphical analysis of the reconstructed WBD datasets based on weighted multilinear regression, using dedicated research tools (Archimede, GE Healthcare, Waukesha, WI, USA). Image-derived input functions were extracted from the descending aorta using an automatic segmentation algorithm to segment an aortic tube of interest (TOI) on an image covering the first few minutes post-injection and then propagated to all dynamic frames. Corrections to the TOI on individual frames, due to inter-frame motion, were taken into account. A time-varying whole-blood to plasma partition coefficient was considered. The sampled plasma input function was modeled by a double exponential covering the WBD part of the input function while for the early continuous part of the input function, an integral preserving linear interpolation was used to generate a discretized final input function. Example frames from the early blood pool single bed dynamic acquisition immediately after injection, together with the estimated IDIF are shown in Figure 1.

### 2.4. Image Analysis

Two physicians (I.A.B. and M.M.), board-certified in nuclear medicine and radiology with 13 and 9 years of experience in diagnostic imaging reviewed the complete imaging data sets of each patient. In consensus, the readers identified target lesions, depicted on standard SUV images, in each patient and defined the lesions as either benign (inflammatory or infection) or malignant. This was completed by reviewing clinical information, histopathology, and all pertinent available imaging data. One reader (Y.P.) measured Ki, Vd, and SUV values: the reader drew volumes of interest around the target lesions as well as standardized volumes into predefined areas for blood pool (aortic arch, 1 cm^3^), bone (fifth lumbar vertebra—L5—2 cm^3^), brain (cerebellum, 2 cm^3^), subcutaneous fat (abdominal wall, 2 cm^3^), liver parenchyma (central, right lobe, 3 cm^3^), lung parenchyma (central, right upper lobe, 3 cm^3^), muscle (gluteus maximus, 2 cm^3^) and spleen (central, 2 cm^3^). The lesions were spatially matched throughout the image sets. In cases of patient’s motion between dynamic (Ki and Vd) and static SUV images, VOIs were manually corrected to represent the same area. Commercial image analysis software (Advantage Workstation Version 4.7, GE Healthcare, Waukesha, WI, USA) was used for the measurements. Lesion-to-background ratios (LBRs) were calculated by dividing target lesion Ki_max_ and SUV_max_ by their respective mean values Ki_mean_ and SUV_mean_ in normal liver tissue similarly to previous studies featuring quantitative SUV analysis [31,32].

### 2.5. Statistical Analysis

All statistical analyses were performed in the open-source statistics software R (version 4.1.0, R Foundation for Statistical Computing, Vienna, Austria) [33]. Categorical variables are expressed as frequency distribution. Continuous variables are presented as mean ± standard deviation if normally distributed or median with interquartile range (IQR) otherwise. Assessment of group differences was determined using an unpaired t-test after ensuring a normal distribution of the data using the Shapiro–Wilk test. For non-normally distributed data, a Wilcoxon test or chi-square test was used. The diagnostic accuracy of parameters differing significantly between benign and malignant lesions was evaluated by calculating sensitivity and specificity at a threshold determined by maximizing Youden’s index area from under the curve from the receiver operating characteristic curve. To assess correlation, Spearman’s rank correlation coefficient ρ was calculated. For all comparisons, a *p*-value of < 0.05 was considered statistically significant.

## 3. Results

### 3.1. Patients’ Characteristics

Twenty-four patients were prospectively included to undergo WBD [^18^F]FDG PET/CT for re-/staging of oncological disease. The most frequent indication for imaging was lung cancer (11/24, 46%) followed by breast cancer (3/24, 13%). The mean age of the participants was 66 ± 16 (47–80) years, the mean BMI was 27.6 ± 6.0 (range 18.8–42.2) kg/m^2^, and the mean injected [^18^F]FDG activity was 210 ± 75 (range 107–303) MBq, as described in Table 1. Example frames from the early blood pool acquisition immediately after the injection and WBD acquisition, together with the estimated IDIF are shown in Figure 1.

### 3.2. Description of Lesions Assessed in the Study Cohort

**Figure 2:**
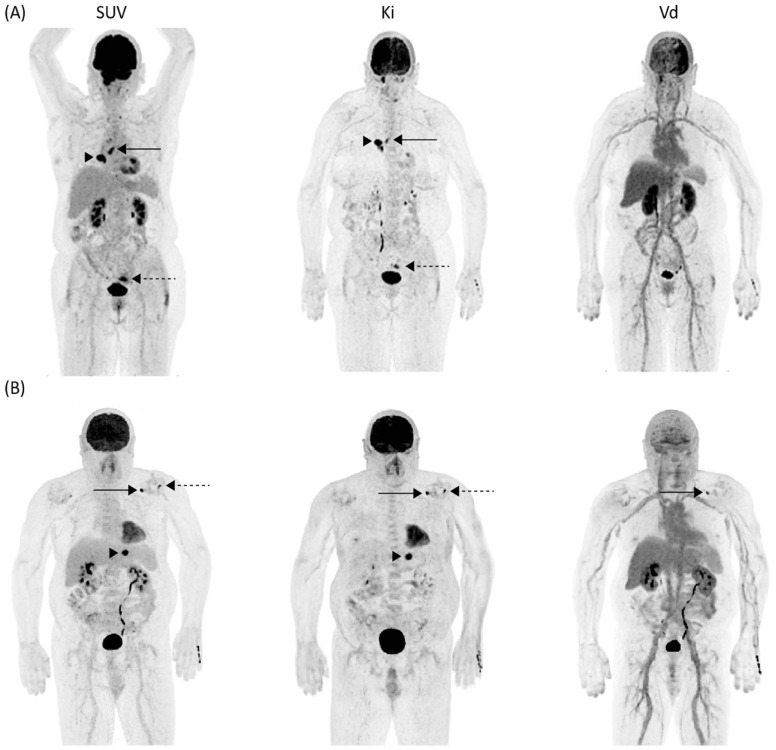
Examples of the parametric images of standardized uptake value (SUV), metabolic rate (Ki) and apparent distribution volume (Vd) of [^18^F]FDG. (**A**) Images from a patient with lung cancer with histologically proven hilar lymph node metastasis, ipsilateral lung metastasis (confirmed by follow-up imaging), and postoperatively proven diverticulitis of the sigmoid colon. High signal is seen in the hilar lymph node (arrow) and ipsilateral lung metastasis (arrowhead) as well as the inflammatory sigmoid colon lesion (dashed arrow) both on SUV and Ki images with no corresponding signal in the Vd image, indicating almost entirely irreversible uptake and no unmetabolized [^18^F]FDG. (**B**) Images from a patient with histologically proven liver metastasis from rectal cancer in the left liver lobe (arrowhead) show high signal in both SUV and Ki images and absence of signal in the Vd image, indicating irreversible uptake of [^18^F]FDG. In the left shoulder, there is one medial lesion (arrow) with high signal on SUV, Ki, and Vd images and one additional lateral focus (dashed arrow) without correlation in the Vd image. Both foci are consistent with tendinitis (confirmed by follow-up imaging), with the medial focus containing partly unmetabolized [^18^F]FDG.

Overall, 77 target lesions were identified. Of these, 60 (78%) lesions were characterized as cancerous, and 17 (22%) as inflammatory or infectious. Table 2 summarizes the distribution of the target lesions by etiology and organ affected. A patient with metastasized lung cancer with hilar lymph node metastasis, ipsilateral pulmonary metastasis, and an incidental [^18^F]FDG avid lesion in the sigmoid colon—postoperatively proven to be chronic diverticulitis—is shown in Figure 2A. Both the cancerous and the inflammatory lesions can be depicted on SUV and Ki maps, but not on Vd maps, suggesting no significant pool of free [^18^F]FDG compared to the surrounding tissues and vasculature. A similar signal’s behavior can be observed in another patient in the segment II liver metastasis from a rectal carcinoma (Figure 2B). Of note, in this patient there was one lesion in the left shoulder with a high signal on both Ki and Vd images attributed to synovitis signifying that not all [^18^F]FDG is metabolized; also, the second shoulder’s focus, consistent with tendinitis on further imaging follow-up, had no uptake on Vd images and was seen only on the Ki image. Overall, compared qualitatively to SUV images, Ki maps show relative suppression of blood pool and parenchymatous organs with high blood fraction and increased contrast of [^18^F]FDG-avid regions.

### 3.3. Quantitative Results of SUV, Ki, and Vd

Quantitative parameters SUV_max_, Ki_max_, and Vd_max_ are summarized according to tissue (blood, bone, brain, cancer, subcutaneous fat, inflammation, lung, muscle, and spleen) in Table 3.

### 3.4. Differentiation of Malignant vs. Inflammatory Lesion in Clinical and Dynamic Imaging

**Figure 3:**
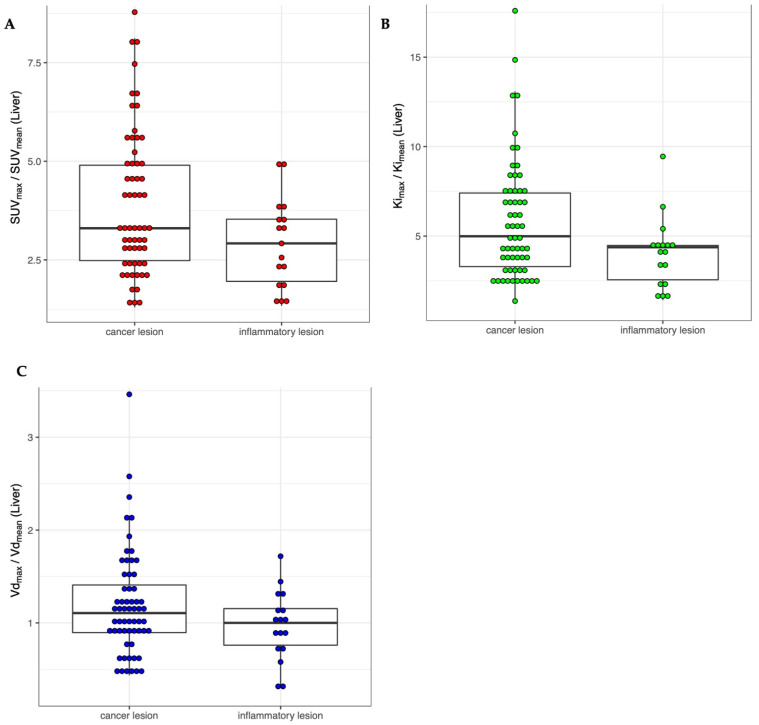
Combined dot and box plots comparing the quantitative parameters standardized uptake value (SUV_max_, **A**), rate of irreversible uptake (Ki_max_, **B**), and apparent distribution volume (Vd_max_, **C**) normalized over their respective mean in liver tissue in cancer (*n* = 60) versus inflammatory lesions (*n* = 17).

**Figure 4:**
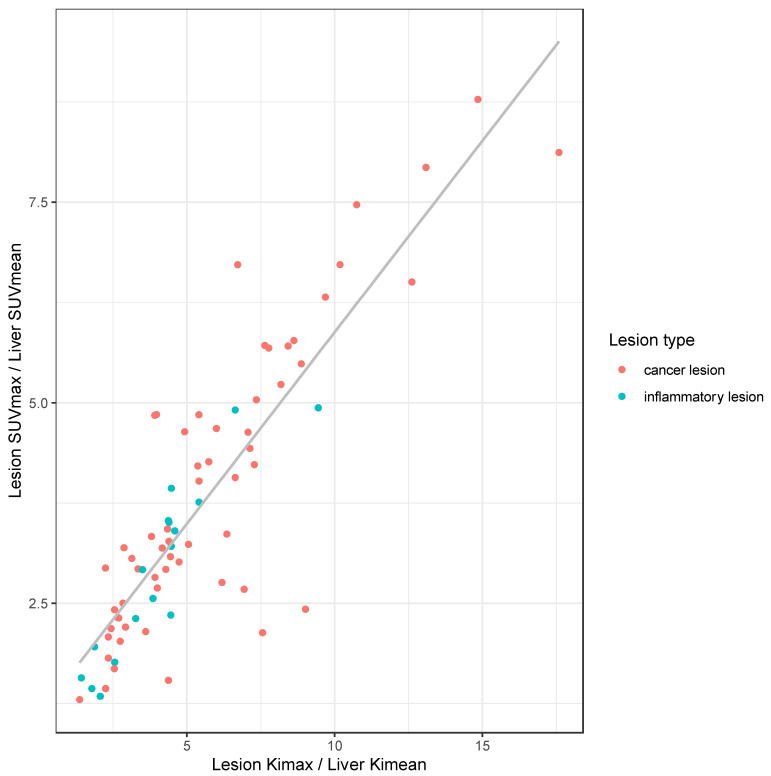
Correlation plot of the rate of irreversible uptake (Ki_max_) against standardized uptake value (SUV_max_). Both parameters were normalized over their respective averages measured in the liver parenchyma.

Quantitative parameters from cancer (*n* = 60) and inflammatory/infectious lesions (*n* = 17), were compared and are given in Table 4. Ki_max_ was significantly higher in cancer lesions compared to infectious or inflammatory lesions (*p* = 0.002). Using a cut-off value for Ki_max_ of 2.6 × 10^−2^ mL/min/mL delivers a sensitivity of 63.3% and a specificity of 82% for the detection of cancer lesions. The LBRs of SUV_max_, Ki_max_, and Vd_max_ against their respective mean values in normal liver tissue did not differ significantly between cancer and infectious or inflammatory lesions (*p* ≥ 0.05), as illustrated in Figure 3. The median LBR of inflammatory and cancer lesions was higher in Ki_max_ than in SUV_max_ (*p* < 0.001). LBRs of Ki_max_ and SUV_max_ showed a strong correlation (ρ = 0.83, *p* < 0.001, Figure 4).

## 4. Discussion

Our study aimed to demonstrate the clinical feasibility of WBD acquisition and explore the value of Patlak macroparameters Ki and Vd for improved differentiation between malignant and inflammatory lesions over the clinically established, static SUV measurements. We demonstrated that WBD step-and-shoot acquisitions are feasible within a clinical setting. In fact, parametric images from Patlak graphical analysis were able to factor out the free [^18^F]FDG, resulting in an increased contrast (LBR), for both inflammatory and malignant lesions. Increased LBR in Ki over SUV images may result in increased detectability of lesions, and previous research has shown equivalent or superior lesions’ detectability for Ki [28]. We did not look specifically at lesion detectability, given that most of our patients suffered from lung cancer and background activity is also very low on SUV images. However, we observed that Ki and Vd were able to improve the specificity by ruling out otherwise false positive findings; as an example, we published a case report about a focus originating from venous collaterals [34].

We found Ki_max_ values to be significantly higher in cancer lesions albeit with poor diagnostic performance for differentiation of cancer and benign lesions, due to a large overlap between the groups. However, we did not observe a significant difference between malignant and inflammatory lesions in their respective LBRs for SUV_max_ and Ki_max_. This contrasts with results from preclinical studies using nude mice with lung cancer and different inflammation models that suggested inflammatory lesions have higher Ki values compared to tumors on dynamic [^18^F]FDG PET [35]. Interestingly, our results showed an opposite trend with higher Ki values for tumors compared to inflammatory/infectious lesions. In fact, in concordance with previous work, we found a significant overlap between malignant and inflammatory lesions without any clear cutoff for accurate differentiation [24]. Furthermore, both cancer and inflammatory or infectious lesions exhibited signals both in the Ki and Vd images. A clear etiology and pattern cannot easily be derived due to the heterogeneous indications and the relatively small number of included patients. More targeted investigations are needed, focusing on specific indications, to identify how specific types of lesions tend to manifest across parametric images, and how vascularity and the free-background [^18^F]FDG affect the parametric lesion’s fingerprint.

In this regard, research on somatostatin analogs with [^68^Ga]DOTATOC and [^68^Ga]DOTATATE has shown Ki to be more closely related to somatostatin receptor expression than SUV, consequently might better reflect therapeutic effect [36,37]. Similar findings have been reported for [^18^F]Fluoride-based assessment of bone metastases from breast cancer, where changes in Ki better reflected the response to therapy [38]. Whether there is a similar role for Ki and [^18^F]FDG for response assessment needs to be elucidated. Data from single bed acquisitions indicate such but have yet to be demonstrated in WBD acquisitions as part of the clinical routine [15,16,17,18]. Furthermore, imaging time in WBD is significantly longer than in static acquisition, thereby limiting the availability of the method overall. If future research can establish a role for additional parameters from WBD, subsequent efforts should be made to determine whether these parameters can be reliably acquired in shorter dynamic acquisitions. To that respect, population-based, instead of individually acquired input functions may play a role.

Our study has limitations. First, the relatively small cohort with a considerable but imbalanced number of cancerous and inflammatory target lesions; also, the relatively wide array of etiologies included, as well as the single-center nature of the study all limit generalizability. Validation across centers and manufacturers should be the subject of further studies. With increasing sample size, quantitative parameters should be analyzed by underlying etiology to assess whether there is a candidate role in certain tumor categories. Second, the target lesions analyzed in our cohort widely lack histopathological confirmation. Due to the nature of our study, it would not have been feasible to obtain histopathologic proof of all lesions included. Lesions have been evaluated by experienced readers carefully, and the designation of all lesions could be performed with high confidence. Third, the acquisition and analysis of WBD datasets are relatively new, and the generation of parametric images is susceptible to noise in the data and patient’s motion. Coupled with the fact that different parts of the body exhibit a variety of kinetics, this warrants individual optimization. Further parameter optimization is desired to reach a point where the benefits of additional parameters become more evident, particularly in clinical tasks where the real underlying benefit is incremental rather than shifting paradigms completely.

## 5. Conclusions

In conclusions, we could show the feasibility of WBD. We found Ki_max_ to be higher in malignant than in inflammatory lesions with poor diagnostic accuracy due to a large overlap between the etiologies. LBRs of Ki_max_ were higher than SUV_max_ with a possible role for enhanced conspicuity of lesions to be elucidated. Whether kinetic parameters can serve as a more precise marker for therapy response or in selected tumor entities needs to be assessed in future studies. Overall, owing to the yet relatively unknown clinical impact of parametric images, further efforts should focus on areas where they are expected to offer the most benefit. Moreover, further workflow improvements, and/or parameter estimation simplifications are necessary for more widespread utilization.

## Figures and Tables

**Table 1:** Demographic data of study subjects (*n* = 24).

Female/male, *n* (%)	8 (33%)/16 (66%)
Age, years	66 ± 16 (47–80)
Body weight, kg	82 ± 25 (55–146)
Body height, m	1.71 ± 0.1 (1.57–1.86)
BMI, kg/m^2^	27.6 ± 6.0 (18.8–42.2)
Blood glucose level at time of injection, mg/dL	107 ± 17 (81–155)
Injected tracer activity, MBq	210 ± 75 (107–303)
Indication for PET	
Lung cancer	11 (46%)
Breast cancer	3 (13%)
Esophageal cancer	2 (8%)
Urogenital cancer	2 (8%)
Head and neck cancer	1 (4%)
Neuroendocrine carcinoma	1 (4%)
Cholangiocarcinoma	1 (4%)
Mesothelioma	1 (4%)
Malignant melanoma	1 (4%)
Lymphoma	1 (4%)

Values are given as absolute numbers and percentages in parenthesis or mean ± standard deviation (range). BMI = body mass index; MBq = Mega-Becquerel; PET = positron emission tomography.

**Table 2:** Distribution of target lesions assessed (*n* = 77).

Lesion Etiology	Lesion Type	Organ	*n*
*Inflammation*			17 (22%)
	Infectious	Pleura	2 (3%)
		Lung	1 (1%)
	Inflammatory	Gastrointestinal tract	5 (6%)
		Lymph node	3 (4%)
		Thyroid	2 (3%)
		Joint	4 (5%)
*Cancer*			60 (78%)
	Primary	Lung	8 (10%)
		Breast	2 (3%)
		Gastrointestinal tract	1 (1%)
		Tonsil	1 (1%)
	Metastasis	Lymph node	16 (21%)
		Bone	10 (13%)
		Lung	8 (10%)
		Pleura	7 (9%)
		Liver	3 (4%)
		Soft tissue	3 (4%)
		Gastrointestinal tract	1 (1%)

**Table 3:** Quantitative parameters maximum standardized uptake value (SUV_max_), maximum rate of irreversible uptake (Ki_max_), and maximum apparent distribution volume (Vd_max_) of various tissues.

	SUV_max_	Ki_max_ [×10^−2^]	Vd_max_
Blood	2.4 (2.0–4)	0.7 (0.4–1.5))	1.0 (0.5–1.5)
Bone	2.5 (1.8–4.7)	1.1 (0.2–2.5)	0.4 (0.2–1.4)
Brain	8.5 (5.5–13.1)	3.1 (0.5–4.4)	1.0 (0.6–1.4)
Cancer	7.8 (3.2–19.1)	3.0 (0.7–11.4)	0.9 (0.3–2.2)
Subcutaneous fat	0.5 (0.1–0.8)	0.2 (0.1–0.9)	0.2 (0.1–0.6)
Inflammation	6.9 (2.6–13.4)	2.0 (0.8–4.2)	0.7 (0.3–1.3)
Liver	3.1 (2.4–4.6)	1.0 (0.6–1.8)	1.1 (0.7–1.5)
Lung	0.8 (0.4–1.6)	0.3 (0.1–0.7)	0.3 (0.1–0.3)
Muscle	1.2 (0.6–1.8)	0.5 (0.1–1.3)	0.2 (0.1–0.4)
Spleen	2.9 (0.1–19.1)	0.8 (0.5–2.2)	0.3 (0.2–0.5)

Values are given as median with range in parenthesis.

**Table 4:** Comparison of quantitative parameters maximum standardized uptake value (SUV_max_), maximum rate of irreversible uptake (Ki_max_), maximum apparent distribution volume (Vd_max_), and lesion-to-background ratio (LBR) between inflammatory/infectious and cancer lesions.

	Cancer Lesions (*n* = 60)	Inflammatory/Infectious Lesions (*n* = 17)	*p*-Value
SUV_max_ [g/mL]	7.8 (IQR 5.9)	6.9 (IQR 2.9)	0.11
LBR (SUV_max_)	3.3 (IQR 2.4)	2.9 (IQR 1.6)	0.06
Ki_max_ [10^−2^ mL/min/mL]	3.0 (IQR 2.2)	2.0 (IQR 1.1)	0.002 *
LBR (Ki_max_)	5.0 (IQR 4.1)	4.4 (IQR 1.9)	0.05
Vd_max_ [mL/mL]	0.9 (IQR 0.5)	0.7 (IQR 0.4)	0.13
LBR (Vd_max_)	1.1 (IQR 0.5)	1.0 (IQR 0.4)	0.18

Values are given as median with interquartile range (IQR) in parenthesis. Statistically significant differences (Wilcoxon test) are marked with an asterisk after the *p*-value.

## Data Availability

The data presented in this study are available on request from the corresponding author.
